# Laparoscopic surgery should be a viable option for T4 colon cancer: evidence from a propensity score matching analysis

**DOI:** 10.1007/s13304-025-02452-2

**Published:** 2025-11-25

**Authors:** Xiaomei Jiang, Hang Zhou, Zhaoyang Zheng, Xiaodong Wang, Zongguang Zhou, Lie Yang

**Affiliations:** 1 Division of Gastrointestinal Surgery, Department of General Surgery, West China Hospital of Sichuan University, No.37 Guoxue Lane, Wuhou District, Chengdu, 610041 Sichuan China; 2 Department of General Surgery, Jiujiang First People’s Hospital, No.48 Taling South Road, Xunyang District, Jiujiang, 332000 Jiangxi China; 3 Institute of Digestive Surgery, State Key Laboratory of Biotherapy and Cancer Center, West China Hospital, Sichuan University, Chengdu, 610041 Sichuan China

**Keywords:** Laparoscopic resection, T4 colon cancer, Safety, Oncological outcomes

## Abstract

**Purpose:**

The suitability of laparoscopy for T4 colon cancer (CC) remains controversial. This study aims to compare the short-term and long-term oncological outcomes specifically for T4 CC.

**Method:**

This observational study included patients who underwent either laparoscopic resection (LR) or open resection (OR) for T4 CC (2015–2023). Propensity score matching (PSM) was used to balance covariates (age, sex, BMI, ASA classification and tumor staging). Primary outcomes were 3-year overall survival (OS) and disease-free survival (DFS). Secondary endpoints included postoperative outcomes and recurrence rates. Prognostic factors for OS and DFS were also analyzed and results of the logistic regression analyses were presented as hazard ratios (HR) with 95 confidence intervals (CI).

**Result:**

A total of 176 patients were enrolled, with 75 well-balanced pairs after PSM. The LR group demonstrated comparable major complication rates to the OR group (8.0% vs. 5.3%, *P* = 0.734). During a mean follow-up of 44.1 ± 28.1 months after LR versus 40.8 ± 23.6 months after OR (*P* = 0.960), postoperative recurrence rates were similar (LR: 24.0% vs. OR: 17.3%, *P* = 0.550). The 3-year OS rates were 76.8% and 81.9% in LR and OR respectively (*P* = 0.292), and 3-year DFS rates were 68.6% and 71.7% in LR and OR, respectively (*P* = 0.312). Multivariate cox regression analysis determined significant independent predictors for OS included age > 75 years (HR = 11.03, 95%CI 5.29–22.98, *P* < 0.001), adjuvant therapy (HR = 0.45, 95%CI 0.23–0.87, *P* = 0.017) and positive lymph nodes (HR = 1.12 per node, 95%CI 1.01–1.25, *P* = 0.035). Key determinants including age > 75 years (HR = 7.25, 95%CI 3.70–14.20, *P* < 0.001), adjuvant therapy (HR = 0.29, 95%CI 0.16–0.53, *P* < 0.001), AJCC staging (III vs. Ⅱ: HR = 2.56, 95%CI 1.07–6.12, *P* = 0.034) and excised lymph nodes (HR = 1.05 per node, 95%CI 1.02–1.07, *P* = 0.001) were indepently associated with DFS.

**Conclusion:**

LR demonstrates comparable short-term and long-term oncological outcomes to OR and it should be considered as a safe and feasible option for T4 CC.

## Introduction

Colorectal cancer is a significant global health challenge, being the third most common malignant tumor and the second leading cause of cancer-related mortality worldwide [1]. Laparoscopic resection (LR) has emerged as a viable alternative to open resection (OR), demonstrating superior short-term outcomes including reduced intraoperative blood loss, shorter hospitalization, and improved cosmesis, while maintaining comparable long-term oncologic safety [2, 3]. Despite established benefits of LR in rectal cancer (COLOR II, COREAN trials), its application in colon cancer (CC) carries unresolved concerns, particularly regarding long-term oncologic safety in T4 lesions. Concerns mainly arises from direct tumor seeding caused by pneumoperitoneum or the operation of laparoscopic instruments, as well as the discrepancy between insufficient operator experience and the need for high technical skill, potentially leading to compromised complete resection rates (R0), increased conversion to open surgery, elevated postoperative complications, and reduced long-term prognosis [4–6].

To date, there is a notable scarcity of robust, evidence-based literature supporting the use of LR in T4 CC. This knowledge gap stems from the inherent technical challenges of T4 tumors, which frequently involve extensive mesenteric infiltration or adjacent organ adhesions, making standardized laparoscopic resection protocols difficult to establish. These complexities contextualize why classic randomized controlled studies, including MRC CLASICC trial, COLOR, Barcelona randomized trials and a landmark multi-institutional study systematically excluded T4 CC [7, 8]. Therefore, the National Comprehensive Cancer Network (NCCN) guidelines generally advise against minimally invasive approaches for this stage of cancer, except in cases where the surgeon has sufficient experience [9, 10].

Given the ongoing debate, this study primarily investigated long-term oncological outcomes, while also placing a secondary emphasis on the comparative analysis of short-term prognostic outcomes between these surgical approaches. Furthermore, key prognostic factors influencing 3-year overall survival (OS) and disease-free survival (DFS) in patients with T4 CC were systematically identified. We hypothesized that LR would be at least as effective as OR. Through a comprehensive analysis, we aimed to identify potential prognostic factors and provide real-world evidence to inform clinical decision-making in the management of T4 CC.

## Materials and methods

### Study design and participants

Our retrospective observational study was conducted at a tertiary referral center and received approval from the West China Hospital Institutional Review Board, Sichuan University. An annual caseload exceeding 500 colon resections with a significant proportion being minimally invasive. Surgical care followed enhanced recovery after surgery (ERAS) protocols and NCCN guidelines. The dedicated surgical oncology team was structured in as follows: minimally invasive surgery division was led by Dr. Lie Yang (Chief Surgeon), who maintains subspecialty certification in minimally invasive techniques. Dr. Yang was primarily responsible for performing all laparoscopic surgeries, having performed more than 3,000 procedures. Open surgery division was directed by Dr. Xiaodong Wang, possessing domain-specific expertise in complex open resections (> 2,000 cases), particularly for locally advanced tumors requiring multivisceral resection. All procedures were carried out in accordance with the ethical principles outlined in the Declaration of Helsinki. A waiver for informed consent was obtained due to the retrospective nature. We enrolled consecutive adult diagnosed with T4 CC from April 2015 to December 2023. Participants were selected according to the following principles:

Inclusion Criteria: (1) patients with CC of pathological stage T4; (2) no distant metastasis; (3) elective curative-intent surgery; (4) American Society of Anesthesiologists (ASA) classification ≤ 4.

Exclusion Criteria: (1) synchronous or metachronous multiple primary tumors or distant metastasis; (2) Emergency surgery; (3) conversion to open surgery; (4) patients lost to follow-up.

Participants were divided into two cohorts: LR and OR. After propensity score matching (PSM) on age, sex, body mass index (BMI), ASA classification, and tumor stage. Baseline, perioperative, and follow-up characteristics were compared between the two groups. The primary outcomes measured were 3-year OS and DFS, while secondary endpoints included postoperative outcomes and recurrence rates.

### Data collection

We collected comprehensive data on patient demographics, comorbidities, surgical details, pathological staging, and follow-up information in *Hospital Information System* of West China Hospital of Sichuan University. Operation modality, gender, age, BMI, diabetes, cardiovascular disease, lung disease, ASA classification, preoperative neoadjuvant therapy, the use of multi-visceral resection, intraoperative blood loss, duration of surgery, total incision length, exhaust time, length of postoperative hospital stay, surgical margins, postoperative adjuvant chemotherapy, operation time, pathologic stage, tumor grade (AJCC staging), number of positive lymph nodes, number of lymph nodes harvested, short-term complications, follow-up duration, recurrence and type of recurrence. 3-year DFS and 3-year OS were both assessed through the use of Kaplan–Meier survival curves.

Surgical margins represent the histologically assessed tumor-adjacent tissue removed en bloc during oncologic surgery. The R classification stratifies resection completeness: R0 (microscopically negative margins) indicates no malignant cells at the inked specimen edges. R1 (microscopically positive) refers to the presence of cancer cells at the edge of the resected tissue. R2 (macroscopically positive) denotes gross residual tumor identified intraoperatively. The Clavien-Dindo (C-D) classification was used to assess the severity of postoperative complications. Complications graded ≥ 3 were considered major, while those graded < 3 were classified as minor complications. 3-year DFS indicated the proportion of patients who, after surgical treatment, remained free of disease recurrence or required further treatment during the survival period. 3-year OS referred to the percentage of patients who were still alive three years post-surgery. Recurrence was defined as either an abnormal increase in tumor markers in blood or other body fluids, excluding other causes, or the identification of a new tumor of the same pathological type as the original tumor at the site of resection or nearby on imaging studies.

### Propensity score matching

To balance the baseline characteristics between the LR and OR groups and minimize selection bias, we performed a 1:1 PSM using the nearest neighbor principle with a caliper of 0.1. Covariates included age, sex, BMI, ASA classification tumor staging and neoadjuvant chemotherapy.

### Data analysis

Continuous variables were expressed as means with standard deviations or medians with the interquartile ranges (IQRs). Categorical variables were expressed as rates or proportions. Chi-square tests or Fisher exact tests were used for categorical variables, and Student’s t-tests and Mann–Whitney U tests were used for continuous variables. Kaplan–Meier methods were employed to analyze the 3-year OS and DFS rates, with log-rank (Mantel-Cox) tests used for inter-group comparisons. Cox regression models were employed to evaluate prognostic factors influencing survival outcomes, with factors deemed significant in univariate analysis subsequently included in the multivariate analysis. Results of the multivariate logistic regression analyses are presented as hazard ratios (HR) with 95% confidence intervals (CI). A p-value of less than 0.05 was considered to indicate a significant between-group difference. All statistical analyses were performed using R Studio for Windows (version 4.3.3; The R Foundation for Statistical Computing, Vienna, Austria).

## Results

### Characteristics of the population

A total of 176 patients were included, with 75 cases in each group after PSM. Baseline characteristics were well-balanced, ensuring comparability (Table 1). In the LR group, 43 females (57.3%) and 32 males (42.7%) were included, compared to 29 females (38.7%) and 46 males (61.3%) in the OR group (*P* = 0.740). The average age was 60.8 ± 12.5 years in the LR group and 63.1 ± 13.1 years in the OR group (*P* = 0.272). Age stratification showed 66 (88.0%) patients ≤ 75 years in the LR group vs. 64 (85.3%) in the OR group (*P* = 0.810). The baseline characteristics remained well-balanced, ensuring comparability between the two groups. BMI was slightly different between groups (LR: 22.4 ± 3.0 kg/m^2^, OR: 23.4 ± 2.8 kg/m^2^, *P* = 0.048, borderline significance), and most patients were ASA II (P = 0.693). No significant differences in comorbidities were observed (*P* = 1.000). Histopathological variables were identical for AJCC staging (Stage II: 62.7% for both groups, *P* = 1.000). In the LR group, 59 (78.7%) cases were pT4a and 16 (21.3%) pT4b; in the OR group, 56 (74.7%) were pT4a and 19 (25.3%) pT4b (*P* = 0.699). Regarding pN staging, both groups had similar distributions (LR: 47 pN0, 17 pN1-3, 11 pN4; OR: 47 pN0, 21 pN1-3, 7 pN4, *P* = 0.520). Neoadjuvant chemotherapy was administered to 26.7% of LR and 25.3% of OR patients, while more OR patients received adjuvant treatment (62.7% vs. 37.3%, *P* = 0.003).

**Table 1:** Patient characteristics after PSM

	Laparoscopic resection (n = 75)	Open resection (n = 75)	*p* value
Gender (F/M)	32 (42.7%)/43 (57.3%)	29 (38.7%)/46 (61.3%)	0.740^c^
Age(YO) (median ± sd)	60.8 ± 12.5	63.1 ± 13.1	0.272^a^
≤ 75	66 (88.0%)	64 (85.3%)	0.810^c^
> 75	9 (12.0%)	11 (14.7%)	
BMI(kg/m^2^) (median ± sd)	22.4 ± 3.0	23.4 ± 2.8	0.048^a^
< 30	74 (98.7%)	74 (98.7%)	1.000^d^
≥ 30	1 (1.33%)	1 (1.33%)	
ASA [n (%)]			0.693^c^
Ⅱ	57 (76.0%)	60 (80.0%)	
Ⅲ	18 (24.0%)	15 (20.0%)	
Comorbidity [n (%)]	15 (20.0%)	14 (18.7%)	1.000^c^
Diabetes	6 (8.0%)	5 (6.67%)	1.000^c^
Cardiovascular diseases	10 (13.3%)	16 (21.3%)	0.281^c^
Pulmonary disease	3 (4.0%)	3 (4.0%)	1.000^d^
Stage of disease AJCC [n (%)]			1.000^c^
Stage II	47 (62.7%)	47 (62.7%)	
Stage III	28 (37.3%)	28 (37.3%)	
pT			0.699^c^
4a	59 (78.7%)	56 (74.7%)	
4b	16 (21.3%)	19 (25.3%)	
pN stage [n (%)]			0.520^c^
0	47 (62.7%)	47 (62.7%)	
1–3	17 (22.7%)	21 (28%)	
4	11 (14.7%)	7 (9.33%)	
Neoadjuvant chemotherapy [n (%)]	20 (26.7%)	19 (25.3%)	1.000^c^
Adjuvant treatment [n (%)]	28 (37.3%)	47 (62.7%)	0.003^c^ **

^a^: Student’s t-test; ^b^: Mann–Whitney U test; ^c^: Chi-Square Test; ^d^: Fisher exact test;

*: *P* < 0.05 (significant difference); **: *P* < 0.01 (highly significant difference); ***: *P* < 0.001 (extremely significant difference)

### Perioperative characteristics

LR had significantly longer operation times (143.9 ± 33.9 vs. 80.8 ± 33.1 min for OR, *P* < 0.001), but both groups had similar median blood loss (20 mL for both, *P* = 0.457). The LR group harvested more lymph nodes (18.2 ± 10.6 vs. 14.3 ± 6.3, *P* = 0.001), with no difference in positive lymph nodes and exhaust time (all *P* > 0.05). The surgical margins of all patients were classified as R0, and this variable was present in 100% of patients in both groups; therefore, no statistical comparison was performed. LR also had a significantly shorter incision length (6.1 ± 2.7 cm vs. 18.8 ± 3.5 cm, *P* < 0.001) and length of postoperative hospital stay (5.9 ± 3.8 d vs. 6.5 ± 1.7 d, *P* = 0.014). (Table 2).

**Table 2:** Perioperative characteristics after PSM

	Laparoscopic resection (n = 75)	Open resection (n = 75)	Statistic	*p* value
Blood loss (ml) [median (IQR)]	20 (37.5)	20 (25.0)	2617.5 ^b^	0.457
Duration of surgery (min) (median ± sd)	143.9 ± 33.9	80.8 ± 33.1	432.0 ^b^	< 0.001 ***
Total incision length (cm) (mean ± sd)	6.1 ± 2.7	18.8 ± 3.5	139.0 ^b^	< 0.001 ***
Exhaust time (d) (mean ± sd)	3.0 ± 1.3	3.0 ± 0.9	3068.5 ^b^	0.302
Length of Postoperative Hospital Stay (d) (mean ± sd)	5.9 ± 3.8	6.5 ± 1.7	2164 ^b^	0.014 *
Adjuvant treatment [n (%)]	47 (62.7%)	28 (37.3%)	8.64^c^	0.003 **
Surgical margins				
R0	75 (100.0%)	75 (100.0%)		1.000
Positive lymph nodes [median (IQR)]	0 (2.0)	0 (1.5)	2755 ^b^	0.805
Harvested lymph nodes (median ± sd)	18.2 ± 10.6	14.3 ± 6.3	1952.5 ^b^	0.001 **

^a^: Student’s t-test; ^b^: Mann–Whitney U test; ^c^: Chi-square test

*: *P* < 0.05 (significant difference); **: *P* < 0.01 (highly significant difference); ***: *P* < 0.001 (extremely significant difference)

### Postoperative outcomes

Table 3 showed that there was no significant difference in complication rates between the LR and OR groups, with 8.0% and 5.3% of patients experiencing major complications, respectively (*P* = 0.743). The most common complication was incision infection, affecting 10.7% of LR patients and 13.3% of OR patients. Other complications included anastomotic leakage (LR: 2.7% vs OR: 4.0%), intestinal obstruction (LR: 4.0% vs OR: 1.3%), abdominal infection (LR: 2.7% vs OR: 1.3%), and pulmonary infection (LR: 1.3% vs OR: 0.0%).

**Table 3:** Short-term outcomes between LR and OR after PSM

	Laparoscopic resection (n = 75)	Open resection (n = 75)	Statistic	*p* value
Complications [n (%)]				
Type	15 (20.0%)	14 (18.7%)	0.000 ^c^	1.000
Anastomotic leakage	2 (2.7%)	3 (4.0%)	1.517 ^d^	1.000
Incision infection	8 (10.7%)	10 (13.3%)	0.777 ^c^	0.802
Pulmonary infection	1 (1.33%)	0 (0.0%)	0.000 ^d^	1.000
Abdominal infection	2 (2.67%)	1 (1.33%)	0.496 ^d^	1.000
Intestinal obstruction	3 (4.0%)	1 (1.33%)	0.327 ^d^	0.620
C-D classification ^#^			0.107 ^c^	0.743
< 3	69 (92.0%)	71 (98.7%)		
≥ 3	6 (8.0%)	4 (5.3%)		

^a^: Student’s t-test; ^b^: Mann–Whitney U test; ^c^: Chi-Square Test; ^d^: Fisher exact test

^#^: C-D classification was Clavien-Dindo classification, used to evaluate the severity of postoperative complications

**Fig. 1:**
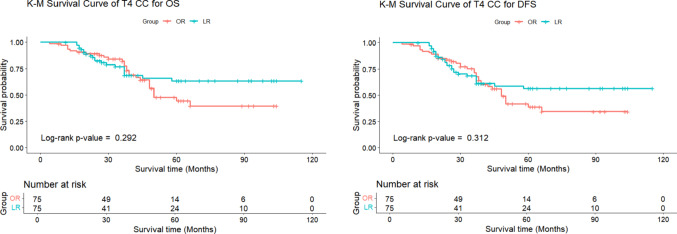
Comparison of 3-year OS and DFS for T4 after PSM

The mean follow-up was 44.1 ± 28.1 months for LR and 40.8 ± 23.6 months for OR (*P* = 0.960). Postoperative recurrence was similar, with 24.0% for LR and 17.3% for OR (*P* = 0.550). Specifically, the peritoneal recurrence rate was 4.0% in both groups (*P* = 1.000). The 3-year OS rates were 76.8% for LR and 81.9% for OR (*P* = 0.292), and DFS rates were 68.6% for LR and 71.7% for OR (*P* = 0.312), with no significant differences between the two groups, supporting the feasibility and safety of the laparoscopic approach (Table 4, Fig. 1).

**Table 4:** Long-term outcomes between LR and OR after PSM

	Laparoscopic resection (n = 75)	Open resection (n = 75)	Statistic	*p* value
Length of follow-up (m) (mean ± sd)	44.1 ± 28.1	40.8 ± 23.6	2826.5 ^b^	0.960
Recurrence [n (%)]	18 (24.0%)	14 (17.3%)	0.358 ^c^	0.550
Local	6 (8.0%)	3 (4.0%)	2.077 ^d^	0.494
Distal	9 (12.0%)	8 (10.7%)	0.000 ^c^	1.000
Peritoneal	3 (4.0%)	3 (4.0%)	1.000 ^d^	1.000
3 year OS (%)	76.8%	81.9%	1.112 ^e^	0.292
3 year DFS (%)	68.6%	71.7%	1.023 ^e^	0.312

^a^: Student’s t-test; ^b^: Mann–Whitney U test; ^c^: Chi-Square Test; ^d^: Fisher exact test; ^e^: log-rank (Mantel-Cox) test

### Univariate and multivariate analysis

Cox proportional hazards regression identified factors influencing survival outcomes (Table 5). For OS, univariate analysis showed significant associations with age (HR: 15.73, *P* < 0.001), adjuvant treatment (HR: 3.07, *P* < 0.001), and positive lymph nodes (HR: 1.13, *P* = 0.037). Multivariate analysis confirmed these findings, with age (> 75 years), adjuvant treatment, and positive lymph nodes as key determinants (HRs: 11.03, 0.45, 1.12, respectively). Notably, older age and positive lymph nodes were associated with poorer OS, and adjuvant treatment might also positively impact OS.

**Table 5:** Factors associated with 3-year OS and DFS after PSM

	OS						DFS					
	Univariate cox regression			Multivariate cox regression			Univariate cox regression			Multivariate cox regression		
	HR	95%CI	*P*-value	HR	95%CI	*P*-value	HR	95%CI	*P*-value	HR	95%CI	*P*-value
Gender (Male VS Female)	0.95	0.53–1.69	0.861				0.79	0.48–1.31	0.362			
Age (> 75 VS ≤ 75)	15.73	7.82–31.67	< 0.001 ***	11.03	5.29–22.98	< 0.001 ***	9.20	4.94–17.13	< 0.001 ***	7.25	3.70–14.20	< 0.001 ***
BMI (≥ 30 VS < 30)	1.08	0.15–7.82	0.941				0.85	0.12–6.12	0.870			
ASA (3 VS 2)	1.64	0.88–3.05	0.119				1.44	0.82–2.54	0.209			
Diabetes	1.65	0.70–3.87	0.253				1.25	0.54–2.91	0.599			
Cardiovascular diseases	1.90	0.99–3.66	0.054				1.52	0.82–2.80	0.184			
Pulmonary disease	2.16	0.77–6.00	0.142				2.12	0.85–5.30	0.107			
Neoadjuvant chemotherapy	1.57	0.84–2.92	0.158				2.27	1.35–3.81	0.002 **	0.66	0.24–1.78	0.411
pT (pT4b VS pT4a)	1.18	0.62–2.27	0.613				1.40	0.80–2.45	0.237			
Blood loss	1.00	0.99–1.00	0.525				1.00	0.99–1.00	0.458			
Duration of surgery	1.00	0.99–1.00	0.585				1.00	0.99–1.01	0.981			
Adjuvant treatment	0.33	0.18–0.59	< 0.001 ***	0.45	0.23- 0.87	0.017 *	0.28	0.16–0.49	< 0.001 ***	0.29	0.16–0.53	< 0.001 ***
Stage of disease AJCC (III VS II)	1.46	0.82–2.60	0.204				2.10	1.27–3.47	0.004 **	2.56	1.07–6.12	0.034 *
pN1-4 (VS pN0)	1.46	0.82–2.60	0.204				2.10	1.27–3.47	0.004 **			
Positive lymph nodes	1.13	1.01–1.27	0.037 *	1.12	1.01–1.25	0.035 *	1.19	1.08–1.30	< 0.001 ***	1.11	0.96–1.28	0.153
Excised lymph nodes	1.01	0.98–1.05	0.464				1.03	1.00–1.05	0.038 *	1.05	1.02–1.07	0.001 **
LR (VS OR)	0.74	0.42- 1.30	0.292				0.77	0.47–1.28	0.313			

*: *P* < 0.05 (significant); **: *P* < 0.01 (highly significant); ***: *P* < 0.001 (extremely significant)

**Fig. 2:**
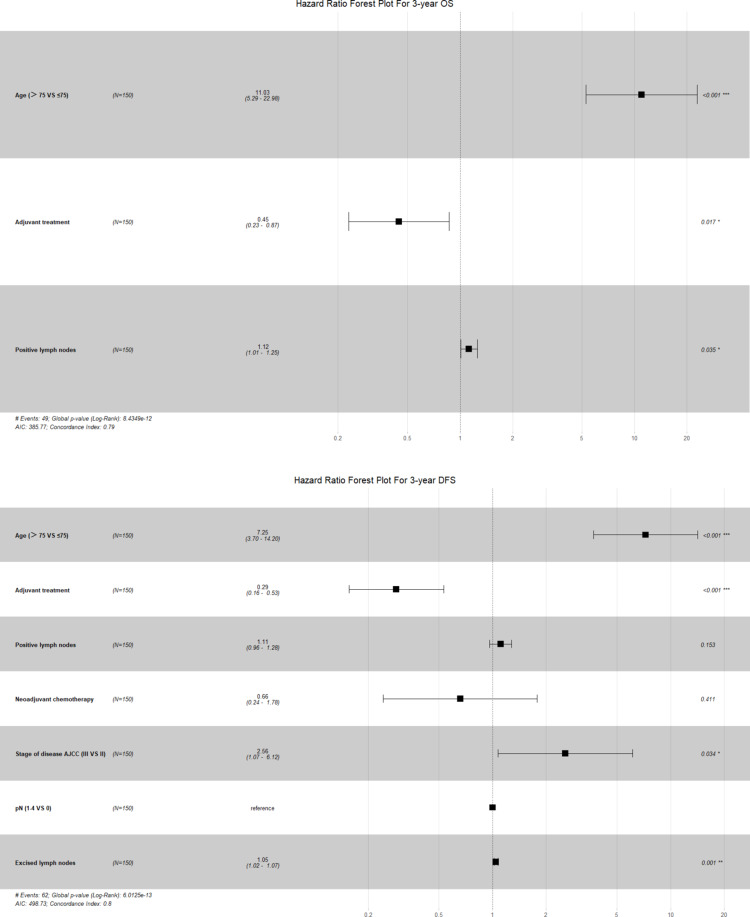
Forest plots of multivariate regression analysis for 3-year OS and DFS

For DFS, univariate analysis revealed associations with age (HR: 9.20, *P* < 0.001), neoadjuvant chemotherapy (HR: 2.27, *P* = 0.002), adjuvant treatment (HR: 0.28, *P* < 0.001), AJCC staging (HR: 2.10, *P* = 0.004), pN1-4 (HR: 2.10, *P* = 0.004), excised lymph nodes (HR: 1.03, *P* = 0.038) and positive lymph nodes (HR: 1.19, *P* < 0.001). Multivariate analysis highlighted age (> 75 years), AJCC staging (III) and excised lymph nodes were significant risk factors (HRs: 7.25, 2.56, 1.05 respectively). Adjuvant treatment was linked to improved DFS, with a HR of 0.29 (Table 5, Fig. 2).

### Subgroup analysis

**Fig. 3:**
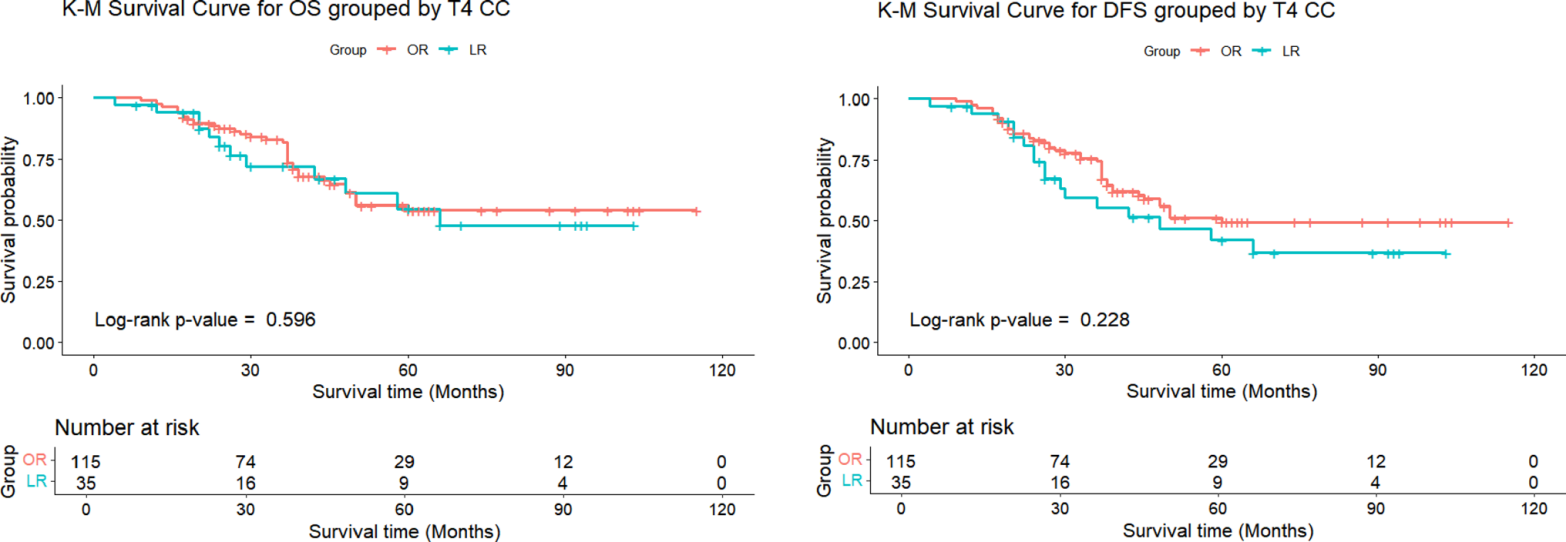
Comparison of 3-year OS and DFS between T4a and T4b CC

After analyzing risk factors, patients were classified into T4a (n = 115) and T4b (n = 35) stages. Outcomes for LR and OR patients did not differ significantly between the stages. The T4a group had fewer complications and lower recurrence rates (19.1%) compared to the T4b group (31.4%). The T4a group demonstrated higher rates of both overall complications (21.7% vs. 11.4%, *P* = 0.268) and major complications (7.8% vs. 2.9%, *P* = 0.454) compared to the T4b group. The average follow-up was 43.1 ± 25.3 months for T4a and 40.2 ± 28.1 months for T4b. Although the T4a group showed a trend towards lower recurrence rates compared to the T4b group (18.3% vs. 31.4%, *P* = 0.083), the 3-year OS (81.6% vs. 72.0%, *P* = 0.596) and DFS (74.4% vs. 55.3%, *P* = 0.228) were numerically higher in the T4a cohort, though none of these differences reached statistical significance (Table 6, Fig. 3).

**Table 6:** Subgroup comparison of outcomes between T4a and T4b CC

	Complications [n (%)]								Length of follow-up (m) (mean ± sd)	Recurrence [n (%)]				3 year OS (%)	3 year DFS (%)
		Type					C-D classification ^#^								
*P*	0.268 ^c^	0.591 ^d^	0.246 ^d^	1.000 ^d^	1.000 ^d^	0.232 ^d^	0.454 ^d^		0.295 ^b^	0.083 ^c^	1.000 ^d^	0.028 ^d^	1.000 ^d^	0.596 ^e^	0.228 ^e^
	Total	Anastomotic leakage	Incision infection	Pulmonary infection	Abdominal infection	Intestinal obstruction	< 3	≥ 3		Total	Local	Distal	Peritoneal		
T4a (n = 115)	25 (21.7%)	5 (4.4%)	16 (13.9%)	1 (0.9%)	3 (2.6%)	2 (1.7%)	106 (92.2%)	9 (7.8%)	43.1 ± 25.3	21 (18.3%)	7 (6.1%)	9 (7.8%)	5 (4.4%)	81.6%	74.4%
T4b (n = 35)	4 (11.4%)	0 (0.0%)	2(5.7%)	0 (0.0%)	0 (0.0%)	2(5.7%)	34 (97.1%)	1 (2.9%)	40.2 ± 28.1	11 (31.4%)	2(5.7%)	8(22.9%)	1(2.9%)	72.0%	55.3%

^a^: Student’s t-test; ^b^: Mann–Whitney U test; ^c^: Chi-Square Test; ^d^: Fisher exact test; ^e^: log-rank (Mantel-Cox) test

^#^: C-D classification was Clavien-Dindo classification

**Table 7:** Respective comparison of tumor outcomes in T4a and T4b CC

	T4a			T4b		
	Laparoscopic resection (n = 59)	Open resection (n = 56)	*P*	Laparoscopic resection (n = 16)	Open resection (n = 19)	*P*
Complications [n (%)]	12 (20.3%)	13 (23.2%)	0.883 ^c^	3 (18.8%)	1 (5.26%)	0.608 ^d^
Type						
Anastomotic leakage	2 (3.4%)	3 (5.4%)	0.674 ^d^	0 (0.0%)	0 (0.0%)	/
Incision infection	7 (11.9%)	9 (16.1%)	0.702 ^c^	1 (6.25%)	1 (5.26%)	1.000 ^d^
Pulmonary infection	1 (1.7%)	0 (0.0%)	1.000 ^d^	0 (0.0%)	0 (0.0%)	/
Abdominal infection	2 (3.4%)	1 (1.8%)	1.000 ^d^	0 (0.0%)	0 (0.0%)	/
Intestinal obstruction	1 (1.7%)	1 (1.8%)	1.000 ^d^	2 (12.5%)	0 (0.0%)	0.202 ^d^
C-D classification ^#^			1.000 ^d^			0.457 ^d^
< 3	54 (91.5%)	52 (92.9%)		15 (93.8%)	19 (100.0%)	
≥ 3	5 (8.5%)	4 (7.1%)		1 (6.3%)	0 (0.0%)	
Length of follow-up (m) (mean ± sd)	44.0 ± 27.5	42.1 ± 22.8	0.669 ^b^	44.3 ± 31.0	36.8 ± 25.8	0.573 ^b^
Recurrence [n (%)]	15 (25.4%)	6 (10.7%)	0.072 ^c^	3 (18.8%)	8 (50.0%)	0.065 ^d^
Local	6 (10.2%)	1 (1.79%)	0.114 ^d^	0 (0.0%)	2 (10.5%)	0.202 ^d^
Distal	6 (10.2%)	3 (5.36%)	0.491 ^d^	0 (0.0%)	5 (26.3%)	0.013 ^d^
Peritoneal	3 (5.08%)	2 (3.57%)	1.000 ^d^	3 (18.8%)	1 (5.26%)	0.608 ^d^
3 year OS (%)	79.2%	83.9%	0.385 ^e^	67.3%	75.7%	0.614 ^e^
3 year DFS (%)	70.5%	78.3%	0.648 ^e^	60.6%	50.9%	0.286 ^e^

^a^: Student’s t-test; ^b^: Mann–Whitney U test; ^c^: Chi-Square Test; ^d^: Fisher exact test; ^e^: log-rank (Mantel-Cox) test

^#^: C-D classification was Clavien-Dindo classification

**Fig. 4:**
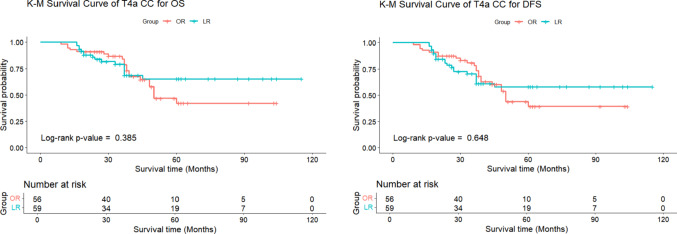
Comparison of 3-year OS and DFS for T4a CC after PSM

**Fig. 5:**
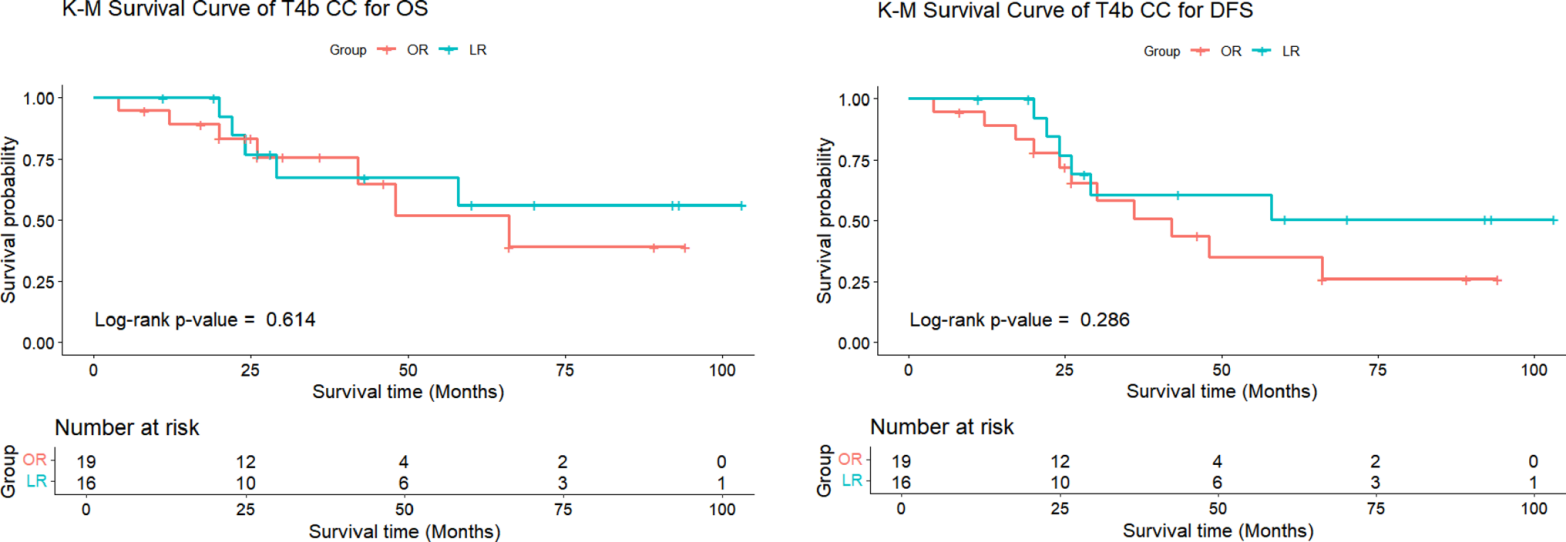
Comparison of 3-year OS and DFS for T4b CC after PSM

In both T4a and T4b patients, no significant differences were observed between LR group and OR group in terms of complications, recurrence, 3-year OS and 3-year DFS. While LR showed a trend toward lower recurrence rates in T4b patients (18.8% vs. 50.0%, *P* = 0.065) and slightly higher recurrence in T4a patients (25.4% vs. 10.7%, *P* = 0.072), none of these differences reached statistical significance. Additionally, the 3-year OS and DFS were numerically higher in the OR group, but these differences were also not statistically significant (Figs. 4 and 5, Table 7).

## Discussion

The introduction of LR for CC has transformed surgical paradigms by offering minimally invasive alternatives with benefits such as reduced blood loss and shorter hospital stays [11, 12]. Nevertheless, there are lack of compelling clinical evidence for T4 CC and persistent disputes existed on correlation between LR and microscopically positive margins [13]. Through rigorous PSM, we demonstrated no significant differences in 3-year OS (76.8% vs. 81.9%, *P* = 0.292) or DFS (68.6% vs. 71.7%, *P* = 0.312) between LR and OR, substantiating LR as a safe and feasible option for selected T4 lesions. These findings challenge prevailing reservations regarding laparoscopic approaches for locally advanced tumors and underscore the viability of LR when performed by experienced surgeons within a structured oncological framework.

### Reconciling safety concerns in T4 CC

The historical reluctance to adopt LR for T4 CC stems from three primary theoretical risks: (1) technical complexity in achieving R0 margins, (2) tumor cell aerosolization induced by CO_2_ pneumoperitoneum, and (3) immune microenvironment alterations potentially favoring residual disease [14, 15]. However, our study demonstrated significantly improved lymph node harvest in the LR cohort (18.2 ± 10.6 vs. 14.3 ± 6.3, *P* = 0.001), attributable to enhanced laparoscopic visualization and surgeon expertise in oncologic dissection [16]. Perioperative outcomes further supported safety equivalence, with comparable intraoperative blood loss [20 (37.5) mL vs. 20 (25.0) mL, *P* = 0.457] and postoperative complication rates [15 (20%) vs. 14 (18.7%), *P* = 1.000] in LR and OR groups. Bang Wool Eom et al. [17, 18] demonstrated that LR itself is not directly associated with complications, which was also proved by our multivariate regression analysis and may be partly attributed to reduced inflammatory stress following LR.

### Minimally invasive characteristics and advantages

Utilizing 5–10 mm trocar incisions versus the 15–25 cm midline laparotomy in OR, LR was associated with less trauma, which correlates with less pain [19]. Notably, a shorter length of hospital stay was observed in the LR group, consistent with findings from previous studies [7]. This benefit is likely due to reduced tissue damage, shorter operative duration, and less postoperative pain. The LOGICA-Trial (2025, n = 225) demonstrated significantly lower opioid consumption in the LR group compared to the OR group during postoperative days (POD) 1–3, with reductions of 131, 118, and 53 mg oral morphine equivalents (OME, mg/day) (all *P* < 0.001), and fewer patients requiring oral opioids at discharge (27% vs. 43%, *P* = 0.006) [20]. Mechanistically, LR also reduced surgical site infections by 58% (2.44% vs. 5.89%, *P* = 0.007). [21]. The study by Allendorf further showed that postoperative cell-mediated detrimental immune function is inversely related to the degree of surgical trauma, potentially through polyclonal activation of CD4 + T cells non-specifically, bypassing the antigen recognition phase and Ana B Martínez-Martínez demonstrated that LR was followed by higher levels of B cells (*P* = 0.023) and lower C-reactive protein levels (*P* < 0.001), comparing to the OR [22, 23]. Importantly, LR does not compromise bowel functional recovery or increase length of postoperative hospital stay, highlighting its remarkable safety.

Mauro Podda et al.[7] conducted a meta-analysis indicating that the mean number of lymph nodes harvested in the LR group was significantly lower than that in the OR group (22.2 ± 5.3 vs. 24.3 ± 5.4, *P* < 0.001). Nevertheless, a greater proportion of patients in the LR group had more than 12 lymph nodes removed.. Several studies have also indicated that the number of lymph nodes harvested was comparable during LR and OR [24]. Our study found that LR resulted in a higher number of harvested lymph nodes. This may be attributed to the magnification provided by laparoscopy, which allows for better visualization of colon lesions and lymph nodes, as well as the operator’s advanced skills and experience accumulated over time.

Notably, adjuvant therapy was more frequently applied after OR. Possible reasons for this phenomenon include: 1). surgical impact: OR often involves more complications and a longer recovery period, which increases the likelihood of recommending adjuvant chemotherapy. In contrast, LR generally leads to quicker recovery, reducing the need for additional treatments. 2). patient health and willingness: patients undergoing OR may have more complex conditions, making them more likely to accept adjuvant therapy. 3). clinician protocols: different medical teams may follow varying treatment protocols depending on the surgical approach. Surgeons may be more inclined to recommend adjuvant therapy after OR due to the invasive nature of the procedure.

### Analysis of long-term efficacy and patterns of tumor recurrence

With a mean follow-up time of 44.1 and 40.8 months, respectively, LR demonstrated equivalent 3-year OS and 3-year DFS compared to OR, consistent with many studies [2, 25]. And this may be attributed to the operator’s proficiency in the laparoscopic learning curve and standardized procedural protocols (e.g., no-touch isolation techniques) [26]. This equivalence persisted despite differences in adjuvant chemotherapy of the LR and OR cohort (62.7% vs. 37.3%, *P* = 0.003), suggesting surgical technique rather than systemic therapy drove outcomes. Regarding recurrence patterns, existing studies have demonstrated that up to 50% of patients with CC experience recurrence or metastasis during the course of their disease [27]. Peritoneal and liver metastases represent the primary causes of mortality in these patients. The observed 21.3% overall recurrence rate in the present study (LR: 24.00% vs. OR: 17.3%, *P* = 0.550) aligns with historical T4 CC benchmarks (2.4%-40.0%).

Peritoneal metastasis, along with liver metastasis, is a major cause of mortality in CC patients and remains a subject of ongoing debate. Studies have shown a high rate of peritoneal recurrence after LR [6, 28]. Nagata et al.[14] suggested that tumor dissemination from pneumoperitoneum, which was accompanied by nebulization of cancer cells, hypothermia and peritoneal acidosis, may contribute to peritoneal metastasis. Additionally, limitations of laparoscopic surgery, such as its reduced ability to detect deep lesions and its smaller scope for irrigation, could leave free-floating cancer cells in the abdominal cavity, increasing the risk of peritoneal recurrence. In our study, the peritoneal recurrence rate was no significant difference in both groups, which can be attributed to the maturity of laparoscopic techniques, strict adherence to tumor-free principles, and effective intraoperative lavage. Additionally, our results are consistent with Pedrazzani et al.[29], reinforcing the non-inferior efficacy of LR compared to OR in complete tumor resection and survival. However, further studies with longer follow-up are needed to confirm this trend and provide more robust data.

Previous studies illuminated the dual biological roles of CO₂ pneumoperitoneum. Intraoperative peritoneal pH monitoring revealed transient acidification, which activates the upregulation and phosphorylation of hypoxia-inducible factor (HIF), enhancing the dissociation of the HIF-1α subunit from HIF-1β, facilitating their interaction with P53, and consequently inhibiting cell apoptosis [30]. Paradoxically, this stress response may also inhibit tumor growth, as evidenced by significantly lower plasma vascular endothelial growth factor (VEGF) levels compared to OR [31]. This dual mechanism necessitates individualized surgical management, as well as further large-scale randomized controlled trials to clarify reciprocal reltionship between surgery, immunity and inflammation on the prognosis.

### Subtype stratification and surgical indications

Subgroup analyses show that LR is less frequently performed in T4b compared to T4a cases, with similar outcomes, likely due to the difficulty distinguishing between inflammatory adhesions and tumor invasion for multi-visceral resection in T4b [32]. Similar results have also been reported by Schootman et al. [26], demonstrating equivalent safety profiles for LR in these two subgroups. Interestingly, T4a may have a higher risk of peritoneal recurrence and poorer prognosis than T4b, possibly because T4b tumors are more likely to be removed en bloc, while T4a tumors spread within the peritoneal cavity [33, 34]. However, T4b cases exhibit significantly higher conversion rates to OR (71.0% vs 12.0%, *P* = 0.003) due to adhesion-related en bloc resection difficulties [35]. Concurrently, they demonstrate worse oncological outcomes: increased lymph node positivity (91.3% vs 69.0%, *P* < 0.001), postoperative recurrence (49.2% vs 27.5%, *P* < 0.001), and reduced R0 resection rates (92.1% vs 97.4%, *P* = 0.012), collectively contributing to inferior 3-year OS (53.1% vs 71.6%, *P* < 0.001) 3-year DFS (DFS:76.7% vs 85.7%, *P* < 0.001) [36]. To sum up, current studies remain limited by small sample sizes yielding for large-scale prospective research to elucidate fundamental differences in tumor biology and prognosis between T4a and T4b CC.

### Contextualizing prognostic factors in evolving evidence

Regarding prognostic factors, advanced age emerged as an independent risk factor for both OS and DFS, consistent with previous studies [37, 38]. The number of positive lymph nodes and excised lymph nodes were significantly associated with OS and DFS, respectively, highlighting the importance of systematic lymphadenectomy. Notably, Chandrakumar et al. [39] pointed out that a smaller number of harvested lymph nodes may lead to an underestimation of the tumor grade. Adjuvant chemotherapy demonstrated a paradoxical association with poorer outcomes [40, 41]. This could potentially be confounded by selection bias: fewer LR patients received chemotherapy (37.3% vs 57.3%). Furthermore, follow-up duration might be potentially insufficient to capture long-term benefits. In contrast to the statistically significant tumor downstaging benefits demonstrated in the landmark FOxTROT trial (NCT00647530)—where neoadjuvant chemotherapy significantly reduced T4 staging prevalence compared to upfront surgery (21.0% vs 31.0%, *P* < 0.001) and improved R0 resection rates (94.0% vs 89.0%, *P* < 0.001)—our analysis showed no survival benefit, likely reflecting regimen completion uncertainty and small cohorts [42]. In particular, pulmonary disease was also associated with reduced long-term outcomes, showing the practical significance of patient screening and individualized assessment.

### Study limitations and future directions

Despite these encouraging findings, our study has several limitations. The retrospective nature of the study and reliance on pathological staging may introduce selection bias. Furthermore, one potential limitation of this study is the exclusion of converted laparoscopic cases. Focusing only on primary laparoscopic procedures may introduce a bias, as the need for conversion could affect the outcomes and benefits of minimally invasive surgery. Additionally, the single-center design limits the generalizability of our results, and larger, multicenter studies are needed to validate these findings. Future research should also focus on identifying additional prognostic factors and optimizing patient selection criteria to further enhance the safety and efficacy of LR in T4 CC.

## Conclusions

In conclusion, our study suggests that LR is a viable and safe alternative to OR for T4 CC, providing comparable short-term and long-term oncological outcomes. These findings offer valuable insights into the decision-making process regarding LR for treating T4 CC. However, careful patient selection and precise surgical techniques remain essential to achieving optimal outcomes.

## Data Availability

The data, materials, and code used in this study are available upon request from the corresponding author.
